# Atezolizumab in combination with carboplatin and etoposide for heavily treated small cell lung cancer

**DOI:** 10.1111/1759-7714.13588

**Published:** 2020-07-24

**Authors:** Nobutaka Kataoka, Yusuke Kunimatsu, Yusuke Tachibana, Takumi Sugimoto, Izumi Sato, Nozomi Tani, Yuri Ogura, Kazuki Hirose, Takayuki Takeda

**Affiliations:** ^1^ Department of Respiratory Medicine Japanese Red Cross Kyoto Daini Hospital Kyoto Japan

**Keywords:** Atezolizumab, carboplatin, etoposide, retreatment, small cell lung carcinoma

## Abstract

Atezolizumab was the first immune checkpoint inhibitor (ICI) to be introduced as a first‐line treatment option for extensive‐stage small cell lung cancer (ES‐SCLC), in combination with carboplatin and etoposide (CE) chemotherapy. However, SCLC treatment options after progression to first‐line chemotherapy are limited, warranting the readministration of previously used drugs. In combination with atezolizumab, CE readministration may theoretically be effective, based on two tentative mechanisms: its additive and synergistic effects on cytotoxic chemotherapy. The additive effect is based on the IFCT‐1603 trial in which the Kaplan‐Meier estimates of both progression‐free survival (PFS) and overall survival (OS) in the atezolizumab group exhibited a tail plateau in the selected population. Conversely, an anti‐PD‐L1 antibody synergistic effect on platinum compounds was assessed in a preclinical study, which was reinforced by clinical data. Thus, atezolizumab in combination with CE may be a treatment option in heavily treated patients. Here, we describe the first case of a heavily treated ES‐SCLC patient treated with chemoimmunotherapy, resulting in a partial response and a durable PFS.

**Key points:**

**Significant findings of the study and what this study adds:**

CE readministration with atezolizumab may be effective based on two tentative mechanisms. Additive and synergistic effects of atezolizumab on CE have been previously suggested via a clinical trial and preclinical study, respectively. This is reflected in the current case in clinical settings.

## Graphical abstract

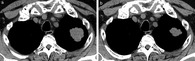

Small‐cell lung cancer treatment options are scarce and limited, which sometimes leads to readministration of previously used drugs in heavily treated patients. Carboplatin and etoposide (CE) readministration with atezolizumab was effective in the current case, which may be based on two tentative mechanisms: additive and synergistic effects of atezolizumab on CE.

## Introduction

The introduction of immune checkpoint inhibitors (ICIs) has revolutionized the treatment strategy of non‐small cell lung cancer (NSCLC) without an oncogenic driver mutation. Atezolizumab, a humanized monoclonal anti‐programmed death ligand 1 (PD‐L1) antibody, was the first ICI to be introduced as an extensive stage small cell lung cancer (ES‐SCLC) first‐line treatment, in combination with carboplatin and etoposide (CE) chemotherapy (IMpower133 trial). In the IMpower133 trial, atezolizumab and CE exhibited a significant improvement over the placebo group, in both overall survival (OS) and progression‐free survival (PFS).^1^ After progression to first‐line treatment, the remaining options are limited to amrubicin, irinotecan, and topotecan therapy. Sometimes, this scarcity leads to the readministration of previously used drugs in heavily treated patients.

Readministration of CE in combination with atezolizumab has not previously been reported. Here, we describe an ES‐SCLC patient in whom atezolizumab plus CE showed a partial response after disease progression, following the second readministration of CE.

## Case report

A 60‐year‐old male ES‐SCLC patient had been previously diagnosed (cT4N3M1a [PUL], stage VIA) 35 months before the start of this study, and had been treated with the following regimens and response evaluations: CE for four cycles with a partial response (PR) and a time to progression (TTP) of nine months, CE rechallenge as a sensitive relapse for four cycles with stable disease (SD) and a TTP of nine months, amrubicin for six cycles with SD, and a TTP of six months with brain metastasis as a progressive disease (PD) site treated using stereotactic radiosurgery (SRS), irinotecan for six cycles with SD and a TTP of six months, and CE rechallenge for the third time administration for two cycles with PD and a TTP of two months.

**Figure 1:**
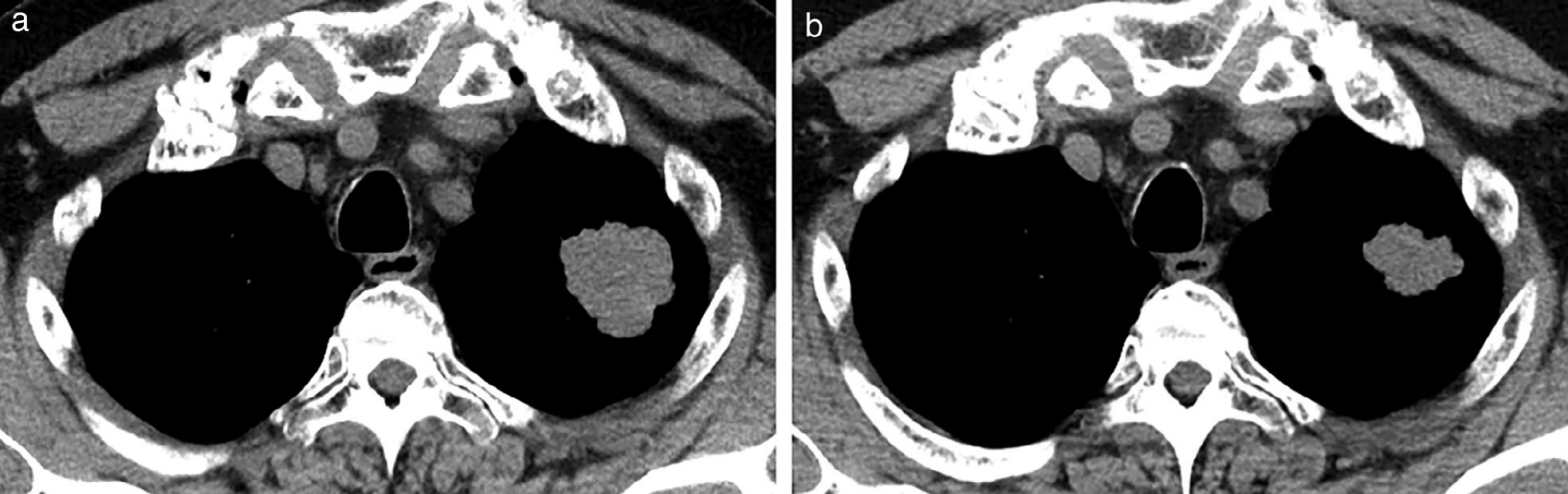
Chest computed tomography in axial slices (**a**) before; and (**b**) after three cycles of chemoimmunotherapy induction with atezolizumab, carboplatin, and etoposide. The primary site located at S^1 + 2^a of the left upper lobe shrunk after treatment, exhibiting a partial response in coronal slices (not shown).

**Figure 2:**
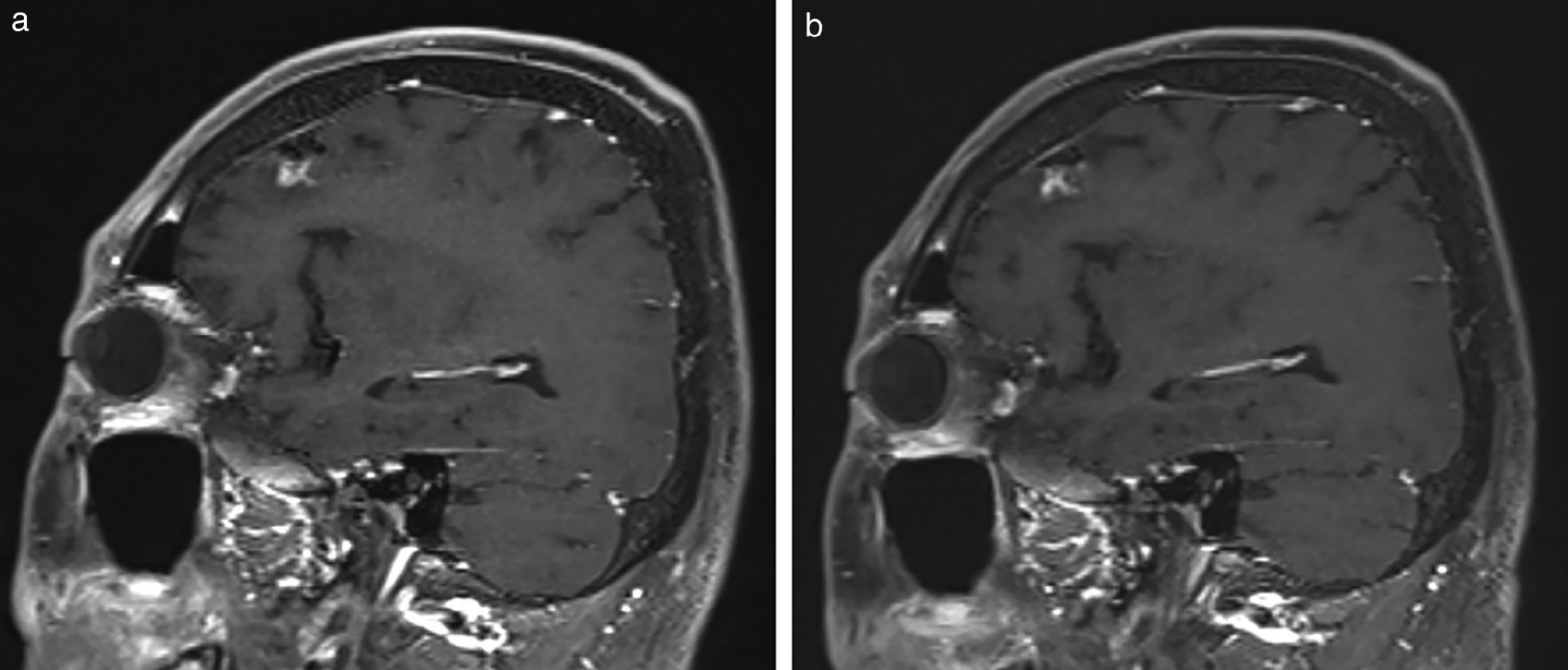
Brain enhanced magnetic resonance imaging (**a**) before; and (**b**) after four cycles of chemoimmunotherapy induction with atezolizumab, carboplatin, and etoposide. The shrinkage of brain metastasis after four induction therapies was limited, with a stable disease.

After three months of treatment holiday following PD with third time CE, atezolizumab and CE were administered, which resulted in a PR after three cycles at the primary site (Fig 1) and in SD with a limited tumor shrinkage of brain metastasis (Fig 2). After four cycles of induction treatment, atezolizumab continuation maintenance therapy was followed for two cycles, and the response remained durable for five months with an ongoing regimen without any immune‐related adverse events.

## Discussion

SCLC is the most aggressive and devastating lung cancer type, which progresses rapidly. However, ES‐SCLC treatment strategies have not changed over the past two decades,^2^ in contrast with NSCLC.

SCLC has diverse tumor suppressor gene targets, and mutations or functional alterations in *TP53*, *RB1*, and *MYC* families genes^3^ pose potential targeted therapies via cellular apoptotic pathway. However, these have not been established as standard SCLC therapies, possibly due to the high sensitivity of SCLC which has previously been reported to initial platinum doublet chemotherapy with OS for 9.3 to 12.8 months,^4^, ^5^, ^6^ which hampers targeted therapies to show superior or noninferior effects over the pre‐existing standard platinum doublet chemotherapy. Therefore, the role of targeted therapies in SCLC may be limited.

Conversely, SCLC frequently develops paraneoplastic syndromes through its potentially high immunogenicity, such as Lambert‐Eaton myasthenic syndrome and limbic encephalitis, or paraneoplastic cerebellar degeneration via several antibodies affecting the neuromuscular junction and central nervous system, respectively.^7^ The potential high immunogenicity of SCLC has been considered a theoretical basis for the introduction of ICIs in SCLC treatment.

Under such circumstances, the IMpower133 trial confirmed atezolizumab and CE efficacy as an ES‐SCLC first‐line treatment.^1^ However, chemoimmunotherapy efficacy on ES‐SCLC is limited in chemotherapy‐naïve ES‐SCLC, and its effect on previously treated ES‐SCLC has not been explored.

The documented efficacy of CE readministration in combination with atezolizumab in the current case may be based on two tentative theories: the additive and synergistic effects of atezolizumab on cytotoxic chemotherapy. The additive effect of atezolizumab in the current case is better understood, as CE readministration immediately before the current treatment resulted in PD. Atezolizumab monotherapy in patients treated with first‐line platinum‐etoposide chemotherapy has previously demonstrated efficacy (IFCT‐1603 trial), in which the response rate and median PFS were 2.3% and 1.4 months, respectively.^8^ Although the IFCT‐1603 trial resulted in a negative result, Kaplan‐Meier estimates of both PFS and OS in the atezolizumab group exhibited a tail plateau, suggesting that there is a selected population who may benefit from SCLC second‐line atezolizumab monotherapy. Conversely, anti‐PD‐L1 antibody synergistic effect on cytotoxic chemotherapy is based on the cancer‐immunity cycle model.^9^ Cytotoxic chemotherapy may accelerate the release of neoantigen, which is a key element of the priming phase in the cancer‐immunity cycle, and changes the immune status from “immune desert” to “immune inflamed” where an ICI can exert its power more effectively.^10^ The synergistic effect of anti‐PD‐L1 antibody on platinum compounds has been assessed in a preclinical study using an NSCLC model, which was reinforced by clinical data elucidating PD‐L1 expression changes in the tumor obtained before and after neoadjuvant platinum‐doublet chemotherapy.^11^ The PD‐L1 expression in SCLC^12^ is relatively low compared with NSCLC, which may also be a limitation of the proposed synergistic mechanism.

Chemoimmunotherapy efficacy on brain metastasis may have been limited due to previous SRS, which decreased viable tumor cells, compared to the primary site. However, chemoimmunotherapy in heavily treated ES‐SCLC may also be effective for brain metastases without SRS.

Considering the above‐mentioned mechanism and atezolizumab and CE efficacy observed in this case, an atezolizumab‐containing regimen may be a treatment option in cases without exposure to anti‐PD‐L1 antibodies.

## Disclosure

The authors declare no conflicts of interest.
